# Review of the use of resource use instruments based on patient recall in relation to other methods of resource use estimation

**DOI:** 10.1186/1745-6215-14-S1-O22

**Published:** 2013-11-29

**Authors:** Colin Ridyard, Dyfrig Hughes

**Affiliations:** 1 Bangor University, Bangor, Gwynedd, UK

## Background

We conducted a review of articles citing trial-related resource use measures catalogued in the Database of Instruments for Resource Use Measurement (http://www.DIRUM.org). The aims were to assess: (i) how the instruments were used in practice; (ii) which items of resource use were most frequently measured using patient-recall; and (iii) how estimates compared if more than one method of data collection was used for the same resource items

## Methods

Articles citing references to measures were identified using Google Scholar, ISI Web of Science and Scopus. These were reviewed and screened according to inclusion criteria. Data were extracted on: the method of administration, resources measured, rates of return and the nature of the other methods of resource use measurement.

## Results

Nearly all citing articles (143/146) used an instrument derived from Beecham and Knapp’s Client Service Receipt Inventory or a variation thereof (81/143). Most measures relied on patient- or proxy-recall (126/146) generally administered during researcher interviews. Use of primary and secondary care was the most widely reported items of resource use (136/146). Twelve studies compared more than one method of data collection for the same resource items and 8/12 showed good correlation between medical records and patient/carer recall or at least indicated one could routinely validate the other. No single administration method was deemed superior.

## Conclusion

Patient interviews are the most common method of questionnaire administration but studies lack clarity on how they are conducted. Results indicate good precision and accuracy in recall questionnaire use; however, concerns about recall bias still exist.

